# Supplementary material for: Subregions in the ventromedial prefrontal cortex integrate threat and protective information to meta-represent safety
Source: PLoS Biol. 2025 Jan 13;23(1):e3002986. doi: 10.1371/journal.pbio.3002986 (PMC11730396; doi:10.1371/journal.pbio.3002986)
Supplement: S1 Text — Mixed effects models predicting safety prediction (probability of choosing “win”) from stimuli safety value, split by stimulus order and type. Table B in S1 Text. Neural response to safety. Significant clusters from group level whole-brain univariate analyses. Table C in S1 Text. Neural response to danger. Significant clusters from group level whole-brain univariate analyses. Fig A in S1 Text. Figure A. Neural response to danger increases during each task phase, highlighting regions of canonical defensive circuitry involved in threat detection such as the insula, thalamus, and PAG. All analyses were conducted using FSL Randomise, TFCE, FWE-corrected p < 0.05. Color bar indicates t-intensity values. (Fig A panels A–C) Parametric increases in whole-brain neural activity that track decrease in experimentally established safety value of stimuli during Danger Prediction. The first stimulus presented represented a bias to partial information, which measures a differentiation in neural activity as a function of stimulus type (threat versus protection). Significant clusters indicate activation increased in those regions as safety probability decreased. Safety decrease was based on the average experimentally established safety probability of each stimulus (protection continuum order: fist, stick, gun grenade; threat continuum order: cat, goose, lion, grizzly). (Fig A panel A) Threat and Protection collapsed, (Fig A panel B) Threat only, (Fig A panel C) Protection only. (Fig A panels D–F) Parametric increases in whole-brain neural activity that track the increased experimentally established safety value of stimuli during Danger Meta-representation. The second stimulus safety value was based on the combined danger probability of the first and second stimuli. For analyses, safety was based on comparison with the average safety value of the stimulus and examined for trials where safety decreased. For example, if a stick was shown as the second stimulus and was paired with a lion [file pbio.3002986.s001.docx]

**Supporting Information**

**Table A.** Mixed effects models predicting safety prediction (probability of choosing ‘win’) from stimuli safety value, split by stimulus order and type.

| **Behavioral**  *N*=100 | Intercept | *B* | *SE* | $\beta$ | *p*  95% CI | AIC  R^2^ |
| --- | --- | --- | --- | --- | --- | --- |
| Protection  First presentation | -2.86 | .76 | .009 | 87.13 | <.001  [.74, .78] | 17534.8  .58 |
| Threat  First presentation | -1.11 | .31 | .005 | 63.83 | <.001  [.30, .32] | 21557.2  .52 |
| Protection  Second presentation | -1.46 | .47 | .007 | 69.51 | <.001  [.46, .49] | 23456.2  .35 |
| Threat  Second presentation | -.01 | .13 | .003 | 47.42 | <.001  [.13, .14] | 26771.5  .22 |
| **MRI**  *N*=30 | Intercept | *B* | *SE* | $\beta$ | *p*  95% CI | AIC  R^2^ |
| Protection  First presentation | -3.08 | .93 | .02 | 46.19 | <.001  [.89, .97] | 4260.86  .69 |
| Threat  First presentation | -2.49 | .43 | .02 | 27.89 | <.001  [.40, .46] | 7842.95  .27 |
| Protection  Second presentation | -1.24 | .54 | .01 | 38.13 | <.001  [.52, .57] | 6352.09  .41 |
| Threat  Second presentation | -.64 | .23 | .01 | 17.81 | <.001  [.20, .25] | 8061.42  .13 |

*Note:* Stimuli scored on a safety scale with 1= danger (stimulus with highest shock probability; grizzly / fist) and 7= safe (stimulus with lowest shock probability; cat / grenade). Danger stimuli scored 1 and 2, Safe stimuli scored 6 and 7.

| **Table B.** **Neural response to safety.** Significant clusters from group level whole-brain univariate analyses. | | | | | | |
| --- | --- | --- | --- | --- | --- | --- |
| Cluster peak region | Voxels | *P*-value | Max Z Value | Peak MNI Coordinates | | |
|  |  |  |  | X | Y | Z |
| **Safety Prediction** | | | | | | |
| All Stim | | | | | | |
| Frontal medial cortex | 124 | 0.005 | 4.01 | 2 | 36 | -16 |
| Lateral occipital cortex | 80 | 0.048 | 3.81 | 42 | -84 | -8 |
| Threat | | | | | | |
| Lateral occipital cortex | 189 | <0.001 | 3.49 | 48 | -68 | -2 |
| Protection | | | | | | |
| Occipital pole | 2286 | <0.001 | 5.81 | -26 | -98 | 0 |
| Lateral occipital cortex | 1306 | <0.001 | 5.8 | 38 | -88 | -6 |
| Middle temporal gyrus | 414 | <0.001 | 4.8 | -64 | -12 | -8 |
| Frontal medial cortex | 302 | <0.001 | 4.39 | 4 | 46 | -16 |
| Central opercular cortex | 200 | <0.001 | 3.98 | -58 | -20 | 10 |
| Temporal occipital fusiform | 156 | <0.001 | 4.51 | 30 | -60 | -12 |
| Subcallosal cortex | 118 | 0.006 | 4.53 | 0 | 8 | -10 |
| Frontal orbital cortex | 103 | 0.013 | 4.47 | -32 | 34 | -16 |
| Cingulate gyrus, anterior | 82 | 0.038 | 4.29 | 0 | 2 | 32 |
| **Safety Meta-representation** | | | | | | |
| All Stim | | | | | | |
| Lateral occipital cortex | 218 | <0.001 | 4.32 | 52 | -72 | -4 |
| Frontal medial cortex | 165 | <0.001 | 3.14 | -12 | 36 | -16 |
| Threat | | | | | | |
| Lateral occipital cortex | 599 | <0.001 | 4.94 | 50 | -74 | -4 |
| Lateral occipital cortex | 319 | <0.001 | 5.26 | -50 | -78 | 2 |
| Lateral occipital cortex | 186 | <0.001 | 4.31 | 32 | -80 | 30 |
| Frontal medial cortex | 128 | 0.003 | 3.87 | 4 | 40 | -20 |
| Protection | | | | | | |
| Occipital fusiform gyrus | 128 | 0.002 | 4.35 | -22 | -80 | -16 |
| Occipital pole | 70 | 0.046 | 4.48 | 14 | -102 | 6 |
| **Safety Recognition** | | | | | | |
| Outcome, Win > Lose | | | | | | |
| Lateral occipital cortex | 13421 | <0.001 | 7.87 | -12 | -62 | 63 |
| Superior parietal lobule | 3722 | <0.001 | 7.68 | -24 | -48 | 73 |
| Precuneous cortex | 601 | <0.001 | 4.44 | -16 | -56 | 19 |
| Superior frontal gyrus | 539 | <0.001 | 6.16 | -8 | 29 | 53 |
| Superior frontal gyrus | 406 | <0.001 | 6.03 | 19 | 33 | 53 |
| Cerebellum | 348 | <0.001 | 6.32 | 1 | -62 | -36 |
| Frontal medial cortex | 250 | <0.001 | 4.58 | -2 | 49 | -10 |
| Middle temporal gyrus | 249 | <0.001 | 4.08 | -66 | -6 | -16 |
| Cingulate gyrus, anterior | 184 | <0.001 | 5.52 | 1 | 17 | 17 |
| Postcentral gyrus | 179 | <0.001 | 4.12 | 63 | -6 | 35 |
| Cingulate gyrus, posterior | 170 | <0.001 | 6.22 | 15 | -26 | 33 |
| Hippocampus | 160 | <0.001 | 6.04 | 21 | -22 | -10 |
| Hippocampus | 152 | <0.001 | 4.74 | -24 | -20 | -14 |
| Precuneous cortex | 98 | 0.001 | 6.79 | 5 | -54 | 59 |
| Thalamus | 92 | 0.002 | 4.37 | 0 | -2 | -6 |
| Superior temporal gyrus | 85 | 0.003 | 3.99 | 69 | -20 | 5 |
| Ventricle | 83 | 0.003 | 8 | 23 | -42 | 19 |
| Planum temporale | 77 | 0.006 | 4.11 | -58 | -18 | 7 |
| Middle temporal gyrus | 71 | 0.009 | 3.93 | 61 | 1 | -18 |
| Ventricle | 64 | 0.016 | 4.5 | 17 | 15 | 19 |
| Ventricle | 63 | 0.018 | 6.34 | 5 | 0 | 21 |
| Planum temporale | 62 | 0.019 | 4.25 | -42 | -34 | 11 |
| Paracingulate gyrus | 56 | 0.032 | 6.07 | 15 | 35 | 21 |
| **Safety Value Updating** | | | | | | |
| High safety, knowledgeable > naive | | | | | | |
| vmPFC (ROI analysis) | 4 | <0.05 | 4.25 | 4 | 18 | -20 |

| **Table C.** **Neural response to danger**. Significant clusters from group level whole-brain univariate analyses. | | | | | | |
| --- | --- | --- | --- | --- | --- | --- |
| Cluster peak region | Voxels | *P*-value | Max Z Value | Peak MNI Coordinates | | |
|  |  |  |  | X | Y | Z |
| **Danger Prediction** | | | | | | |
| All Stim | | | | | | |
| Occipital pole | 230 | <0.001 | 5.22 | -10 | -90 | -6 |
| Postcentral gyrus | 137 | 0.003 | 4.65 | -42 | -24 | 50 |
| Threat | | | | | | |
| Occipital pole | 1428 | <0.001 | 5.91 | -12 | -94 | -8 |
| Postcentral gyrus | 423 | <0.001 | 5.31 | -42 | -24 | 50 |
| Lingual gyrus | 407 | <0.001 | 4.97 | 8 | -86 | -12 |
| Occipital fusiform gyrus | 297 | <0.001 | 5.23 | 34 | -82 | -16 |
| Temporal occipital fusiform | 132 | 0.004 | 4.28 | -28 | -54 | -16 |
| Cerebellum | 115 | 0.008 | 3.88 | 34 | -52 | -24 |
| Protection | | | | | | |
| — | — | — | — | — | — | — |
| **Danger Meta-representation** | | | | | | |
| All Stim | | | | | | |
| Parietal operculum cortex | 871 | <0.001 | 12.7 | 44 | -20 | 18 |
| Thalamus | 850 | <0.001 | 12.4 | 6 | -18 | -4 |
| Insular cortex | 635 | <0.001 | 4.72 | 34 | 14 | 8 |
| Superior frontal gyrus | 620 | <0.001 | 9.78 | 2 | 30 | 52 |
| Parietal operculum cortex | 536 | <0.001 | 8.73 | -52 | -26 | 20 |
| Frontal orbital cortex | 409 | <0.001 | 6.92 | -38 | 24 | -4 |
| Caudate | 251 | <0.001 | 4.65 | -10 | 6 | 2 |
| Occipital pole | 160 | <0.001 | 3.05 | -8 | -96 | -4 |
| Cerebellum | 159 | <0.001 | 3.03 | -34 | -60 | -28 |
| Cingulate gyrus, posterior | 127 | 0.004 | 4.21 | -4 | -24 | 26 |
| Threat | | | | | | |
| Occipital pole | 706 | <0.001 | 6.31 | -12 | -94 | -8 |
| Occipital pole | 227 | <0.001 | 4.49 | 14 | -100 | 0 |
| Thalamus | 147 | 0.001 | 4.31 | 4 | -16 | -4 |
| Superior frontal gyrus | 123 | 0.004 | 4.17 | 4 | 34 | 52 |
| Frontal orbital cortex | 79 | 0.041 | 3.89 | 30 | 28 | -2 |
| Protection | | | | | | |
| Frontal orbital cortex | 491 | <0.001 | 4.99 | 34 | 22 | -14 |
| Parietal operculum cortex | 324 | <0.001 | 4.34 | 46 | -30 | 22 |
| Supramarginal gyrus | 255 | <0.001 | 3.97 | 62 | -42 | 30 |
| Intracalcarine cortex | 224 | <0.001 | 3.95 | -12 | -82 | 6 |
| Intracalcarine cortex | 193 | <0.001 | 4.37 | 14 | -86 | 6 |
| Thalamus | 128 | 0.002 | 3.82 | 10 | 0 | 8 |
| Frontal orbital cortex | 126 | 0.002 | 4.3 | -38 | 22 | -6 |
| Parietal operculum cortex | 112 | 0.004 | 3.98 | -58 | -40 | 24 |
| Cingulate gyrus, anterior | 94 | 0.011 | 3.95 | 2 | 6 | 42 |
| Middle frontal gyrus | 84 | 0.02 | 4.19 | 42 | 2 | 52 |
| Cingulate gyrus, anterior | 69 | 0.049 | 3.82 | 8 | 26 | 28 |
| **Danger Recognition** | | | | | | |
| Outcome, Lose > Win | | | | | | |
| Frontal pole | 6974 | <0.001 | 6.46 | 55 | 43 | 0 |
| Caudate | 2273 | <0.001 | 6.02 | -10 | -6 | 17 |
| Cingulate gyrus, anterior | 1970 | <0.001 | 6.23 | -10 | -10 | 43 |
| Supramarginal gyrus | 1618 | <0.001 | 5.26 | -62 | -40 | 33 |
| Middle frontal gyrus | 496 | <0.001 | 6.14 | 49 | 35 | 35 |
| Frontal pole | 344 | <0.001 | 4.63 | 19 | 45 | 19 |
| Lingual gyrus | 241 | <0.001 | 5.03 | -6 | -76 | 3 |
| Thalamus | 227 | <0.001 | 6.18 | 17 | -24 | 1 |
| Precuneous cortex | 179 | <0.001 | 4.81 | 9 | -74 | 39 |
| Cerebellum | 145 | <0.001 | 4.34 | -22 | -70 | -30 |
| Cerebellum | 135 | <0.001 | 5.63 | -36 | -54 | -60 |
| Thalamus | 104 | <0.001 | 5.41 | -14 | -26 | -6 |
| Frontal pole | 71 | 0.009 | 3.83 | -38 | 41 | 19 |
| Precuneous cortex | 70 | 0.01 | 3.84 | -14 | -68 | 35 |
| Superior parietal lobule | 70 | 0.01 | 4.26 | 17 | -48 | 61 |
| Putamen | 64 | 0.016 | 4.83 | 23 | 1 | -2 |
| Temporal fusiform cortex | 55 | 0.035 | 6.68 | 43 | -12 | -32 |
| Cerebellum | 54 | 0.039 | 6.69 | 21 | -70 | -56 |
| Brain stem | 52 | 0.046 | 5.05 | 9 | -40 | -18 |
| **Danger Value Updating** |  |  |  |  |  |  |
| High danger, knowledgeable > naive |  |  |  |  |  |  |
| — | — | — | — | — | — | — |

**
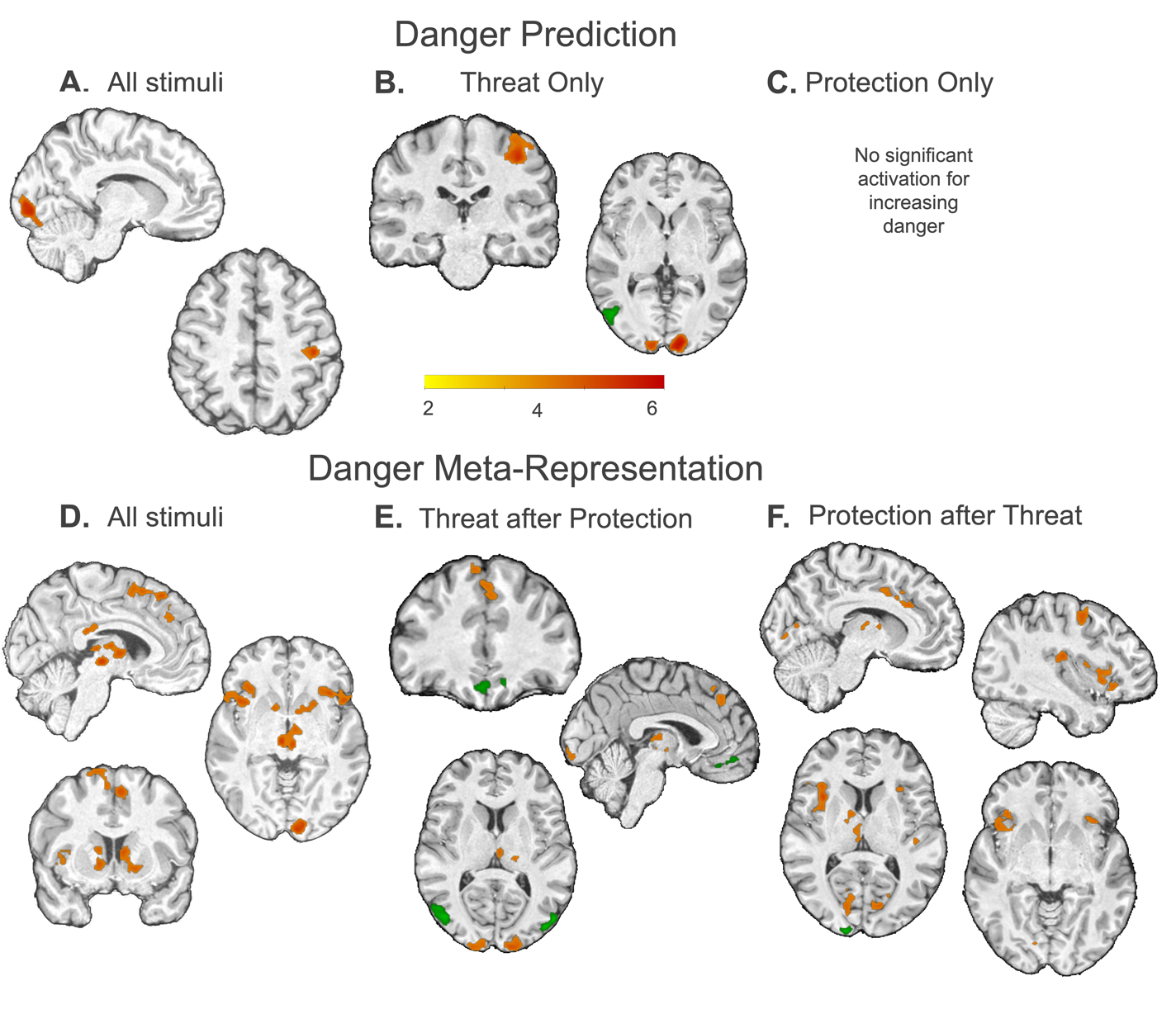
**

**
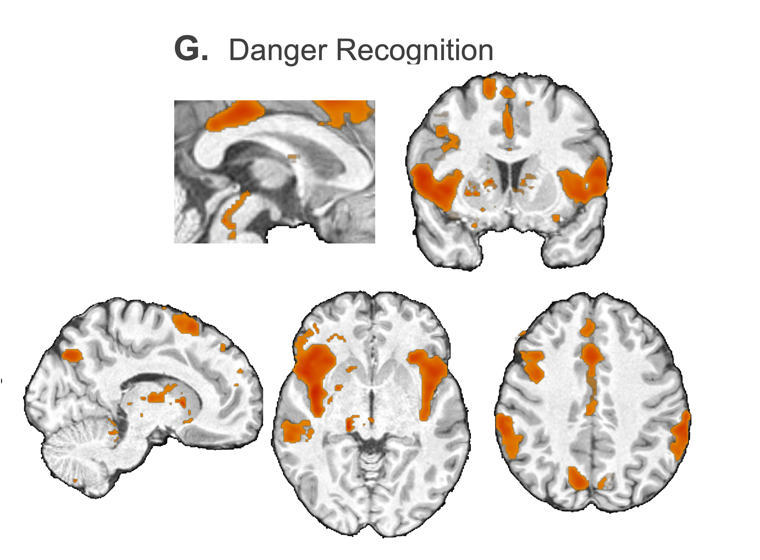
**

**Figure A. Neural response to danger increases during each task phase, highlighting regions of canonical defensive circuitry involved in threat detection such as the insula, thalamus, and PAG.** All analyses were conducted using FSL Randomise, TFCE, FWE-corrected *p*<.05. Color bar indicates t-intensity values. **(Fig. A panels A-C)** Parametric increases in whole-brain neural activity that track decrease in experimentally established safety value of stimuli during **Danger Prediction**. The first stimulus presented represented a bias to partial information, which measures a differentiation in neural activity as a function of stimulus type (threat versus protection). Significant clusters indicate activation increased in those regions as safety probability decreased. Safety decrease was based on the average experimentally established safety probability of each stimulus (protection continuum order: fist, stick, gun grenade; threat continuum order: cat, goose, lion, grizzly). (Fig. A panel A) Threat and Protection collapsed, (Fig. A panel B) Threat only, (Fig. A panel C) Protection only. **(Fig. A panels D-F)** Parametric increases in whole-brain neural activity that track the increased experimentally established safety value of stimuli during **Danger Meta-representation.** The second stimulus safety value was based on the combined danger probability of the first and second stimuli. For analyses, safety was based on comparison with the average safety value of the stimulus and examined for trials where safety decreased. For example, if a stick was shown as the second stimulus and was paired with a lion, the probability of safety would reduce from 35.72% (safety average for all stick trials) to 21.43% (safety when stick is paired with lion) (see Figure 1B). **(Fig. A panel D)** Threat and Protection collapsed, **(Fig. A panel E)** Threat only, **(Fig. A panel F)** Protection only. **(Fig. A panel G)** Neural activation in response to **Danger Recognition** when subjects learned they were unsuccessful in battle. Analyses probed response at the outcome screen when it indicated potential for electric shock (20%) compared to when it indicated certain safety from shock (100%). Task images are approximate reproductions. Credit: grizzly: iStock.com/jhorrocks, lion: iStock.com/Wirestock, goose: iStock.com/MikeLane45, cat: iStock.com/GlobalP, grenade: iStock.com/vuk8691, gun: iStock.com/goir, stick: iStock.com/happyfoto, fist: iStock.com/bbbrrn. Source data can be found at <https://osf.io/8qg7y/> under ‘MRI data’.

**A.**

**B.**

**C.**

**D.**

**Fig B. Results of stimuli development** **and selection of stimuli at the high and low ends of the safety estimation spectrum.** Two questions were asked related to level of danger (animals) and power (weapons) of 20 potential stimuli images. Items were also paired in head-to-head battles with all other stimuli of the same type. Lion and grizzly were rated as the most dangerous stimuli and cat and goose were rated as the second and third least dangerous stimuli (rat was selected as the least dangerous but ultimately excluded from the set to avoid conflating threat with disgust). The same rankings were reported for the head-to-head battles across all animals. The grenade and gun were rated as the most powerful weapons and as most likely to win head-to-head. Fist and stick were rated in the bottom 30% of power ratings and bottom 20% of head-to-heads. Other weapons rated as less powerful were excluded due to concerns of unwieldy usage (i.e., rope). Simulus design was inspired by 2021 YouGov survey of 1224 adults showing that that 6% of Americans believe they could beat a grizzly bear in a fight without weapons (retrieved from: https://today.yougov.com/society/articles/35852-lions-and-tigers-and-bears-what-animal-would-win-f). Animals were selected as pilot stimuli based on those survey results and a selection of weapons across the range of potential danger was also tested. Animal images were selected to be ‘attacking’ to mitigate any issues of liking the animal (cuddly housecat versus angry housecat) to ensure stimuli were immediately recognizable as a threat. Weapon images were selected to be without any other stimuli in the picture and on white backgrounds. All images were kept in black and white and sepia tone ranges.
