# Supplementary material for: Subregions in the ventromedial prefrontal cortex integrate threat and protective information to meta-represent safety
Source: PLoS Biol. 2025 Jan 13;23(1):e3002986. doi: 10.1371/journal.pbio.3002986 (PMC11730396; doi:10.1371/journal.pbio.3002986)
Supplement: Attachment — Submitted filename: PBIOLOGY-D-24-02409R1_ResponsetoReviewers_Nov28.docx [file pbio.3002986.s002.docx]

**PBIOLOGY-D-24-02409R1**

**EDITOR COMMENTS:**

**E1.** Among the concerns raised, we would like to highlight the need to (i) address Reviewer 1's comment to map the different sub-regions of vmPFC to any specific anatomical boundaries and speculate on why these different portions were involved in the different processes, (ii) more carefully define and introduce the concepts and how they relate to your study, and (iii) provide evidence that the task did engage the constructs and processes it is supposed to engage and that the fMRI sequence is suitable.

**AE1. We have now addressed the mapping of different subregions of the vmPFC to anatomical boundaries. We included a ‘Key Concepts’ section to aid in defining and introducing the concepts. We provided detailed information about the MRI sequences to confirm they are suitable. We responded to R3’s concern about construct validity with evidence of tasks evoking the processes intended.**

**REVIEWER COMMENTS:**

**Reviewer #1 (Patricia Lockwood):** This is an exciting, novel and rigorously conducted study that I enjoyed reading. The behavioural and neural mechanisms of safety and protection remain poorly understood but are crucial drivers of human valuation and decision-making. The combination of a behavioural study with an fmri study is impressive, and studies were preregistered. The use of real shock stimuli is challenging to implement but adds ecological validity to the task. The analyses are sophisticated including univariate and multivariate approaches as well as connectivity analyses. The conclusions are balanced and appropriate. I have just a few comments the authors may wish to consider.

**We sincerely thank the Reviewer for their support for this work and appreciate their suggestions for improvement, which we have addressed below.**

**R1.1.**      It is very interesting to find different sub-regions of vmPFC involved in different aspects of the task. I wondered if the authors could map these to any specific anatomical boundaries and speculate on why these different portions were involved in the different processes. In light of this I would also hesitate to suggest that these portions represent a gradient but rather different portions of vmPFC.

**R1.1. Thank you for this suggestion, we think being clearer about the anatomical boundaries for our findings will improve the interpretation and impact of this work. The vmPFC findings from our parametric modulation results (Figure 3a, c, d, e) are present in area 14m as outlined by Mackey, S., & Petrides, M. (2014). Architecture and morphology of the human ventromedial prefrontal cortex. *Eur J Neurosci*, *40*, 2777–2796.**

**Some activation from safety recognition and safety updating extend into other regions of the vmPFC, namely Area 10.**

**Given that our safety-related vmPFC findings primarily occur within 14m we have retained the ‘gradient’ language. Mackey & Petrides note possible subparcellation of area 14m into subareas with posterior to anterior distinction such that the posterior part of area 14m is less well developed and is less granular than the anterior part. We note that most of our activation, particularly that with increasing safety identification during recognition and updating, is in anterior 14m.**

**We now specify more clearly these anatomical distinctions in the results:**

**“*Safety activation in the vmPFC is primarily identified in area 14m with safety updating and recognition (conditions during which safety was guaranteed) extending to area 10 (frontal pole).^34^”***

**and discuss their potential significance in the discussion:**

***“The vmPFC coded safety during all task states from Safety Prediction to Meta-representation to Safety Recognition and Value Updating. Activation to these states was primarily identified in area 14m, with Safety Recognition and Value Updating extending to area 10. We identified what appeared to be a gradient from the posterior to the anterior part of vmPFC area 14m, with activation extending more anterior as safety increased in certainty. This apparent gradient supports the possibility of subparcellation of area 14m into smaller areas with posterior and anterior distinction. During detailed mapping of vmPFC architecture, Mackey & Petrides (2014) note that “the posterior part of area 14m is less well developed and is less granular than the anterior part”.^34^ This aligns with our recent proposition^2^ that the posterior vmPFC may encode simpler representations of threat whereas the anterior vmPFC may encode more complex safety associations. This apparent gradient aligns with the broader organizational gradient of the prefrontal cortex, where anterior regions are generally more granular and complex, reflecting their role in higher-order cognitive and integrative functions. Our findings support assertions that the anterior vmPFC plays a role in integrating the value of self-relevant stimuli to influence the higher-order construction of affective processes, including safety.^2,61^* ”**

**In response to Reviewer 3 we now clarify the vmPFC ROIs selected for Informational Connectivity analysis – the ‘anterior vmPFC’ ROI was identified as the ‘frontal medial cortex’ and the ‘posterior vmPFC’ ROI was identified as the ‘subcallosal cortex’, both from the Harvard Oxford cortical atlas: “*Regions of Interest (ROIs). ROIs were used in two analyses, multivariate searchlight, and multivariate informational connectivity. All other analyses were conducted using the whole brain. ROIs were independently defined based on analytic purposes. First, a large vmPFC mask was independently defined using Neurovault and applied as a small-volume correction for the Safety Value Updating searchlight analysis. The vmPFC mask (***[***https://identifiers.org/neurovault.image:132836***](https://identifiers.org/neurovault.image:132836)***) consisted of 152 subjects and 4233 voxels. The mask was transformed to standard space (MNI152NLin2009cAsym) using ‘flirt’ [FSL 6.0.5.1:57b01774, @flirt]. A large vmPFC mask was used to assess whether different subregions of the vmPFC were implicated in Safety Value Updating rather than constraining search to a specific subregion. Second, ten separate ROIs were defined for Multivariate Informational Connectivity analyses. Regions were selected based on threat acquisition and extinction circuitry in prior literature and defined using the Harvard-Oxford cortical and subcortical structural atlases, except for the PAG which was defined using Neurosynth meta-analysis (https://neurosynth.org/; “periaqueductal” with an association test). Instead of using the larger homogenous vmPFC structure as constrained during searchlight, we divided the vmPFC into anterior and posterior subparts based on the Harvard-Oxford atlas to examine distinct connectivity within these regions. The vmPFC ROIs used in Informational Connectivity analyses were contained within the vmPFC mask used in the searchlight. The ten ROIs selected were the anterior vmPFC (‘frontal medial cortex’), posterior vmPFC (‘subcallosal cortex’), thalamus, ACC, bilateral insula, dorsal striatum, ventral striatum, PAG, bilateral amygdala, bilateral hippocampus.”***

**R1.2.**      I wonder how the authors see their results fitting with the work on self prioritisation and self relevance in vmPFC? (e.g. Sui & Humphreys, 2015; Sui & Gu., 2017; Lockwood et al., 2018). As several studies have shown that self relevant, as opposed to stranger and friend relevant information, is preferentially encoded in vmPFC. 

**A1.2. We greatly appreciate these citation suggestions and agree that our study fits with self-prioritization and relevance in the vmPFC. We’ve added to the introduction and discussion to better integrate this work with the hope that it improves the contribution of our findings to the social cognition literature.**

**“*We hypothesize a candidate region for human safety coding is the ventromedial prefrontal cortex (vmPFC). Recent theoretical developments suggest representations of threat and protective information are encoded in canonical defensive and cognitive neural circuits, the latter including the vmPFC.^2,17–22^ The vmPFC aids the affective processing of safety signals as well as the acquisition of new threat associations and threat extinction during Pavlovian threat conditioning.^2,17–28^ With relevance to the current study, we propose that external threats are computed in a bottom-up fashion, primarily driven by sensory processing regions of the brain, whereas protection is integrated with threat information through top-down metacognitive circuitry related to self-evaluation, including the vmPFC.^29,30^ ^31^Work outside of the threat context points to the vmPFC as pivotal in supporting decisions related to the self^16^ and in showing bias toward information concerning the self.^31^ The vmPFC contributes to binding self-relevant information across cognitive domains such as perception, memory, and decision-making, thereby enhancing the integration of stimuli with self-representations.^32^ Research on threat controllability provides links between self-referential cognition and safety – controllability, which relies on self-referential processing, improves fear extinction, which reflects safety, in humans, thus implying a role of the self in safety estimation.^332,17–28^Consequently, we identify the vmPFC is a potentially important region for integrating self and external information to formulate safety estimations.*”**

**“*Despite equivalent experimentally established safety probabilities for threat and protection stimuli, only protection evoked activation in the vmPFC. One intriguing possibility for vmPFC sensitivity to protective weapons, beyond their safety relevance to the self, is that the vmPFC was coding ownership associations between objects and the self.^31^ We did not directly test whether ownership was part of the cognitive milieu in our study, but our findings are consistent with the perspective that the vmPFC assigns personal significance to objects based on its meaning and function for the self.^35^ Our findings agree with prior literature establishing that the vmPFC tracks self-related associations beyond reward-based learning^35^ and point to a specialized role of the vmPFC in tracking self-relevant information during safety estimation.”***

***“Our findings converge with prior work indicating the vmPFC is involved in updating mental representations of threat and reward particularly in contexts like fear extinction.^17^ Given vmPFC involvement in the physiological regulation of autonomic processes like heart rate and skin conductance,^42,43^ it is likely that vmPFC engagement we saw during our task reflects a more complicated myriad of processes that we did not explicitly measure, including emotion regulation.^44,4545^ Additionally, our behavioral results show safety recognition varied as a function of stimulus type, and the vmPFC was preferentially activated to protection during the Safety Estimation Task. These findings are consistent with the vmPFC playing a role in self-relevant processes specifically rather than undifferentiated emotion regulation broadly. If the vmPFC were primarily tracking the latter, we would expect to see consistent activation regardless of stimulus type because both stimuli presentations occur during a need for regulatory engagement. Our findings fit with an interpretation of the vmPFC as providing a substrate for linking experiences, contexts, and events to the bio-regulatory or emotional state of the self.^32,46^”***

***“Multivariate connectivity revealed a safety network consisting of the anterior and posterior vmPFC, dorsal and ventral striatum, caudodorsal ACC, and insula. The hippocampus, thalamus, and amygdala also emerged as connected to this core network, but with less consistency depending on stimulus relevance. In response to Safety Prediction, threat and protection networks showed a shift in hub organization from the posterior to anterior vmPFC. The dorsal striatum emerged as a core hub for Safety Meta-representation. The dorsal striatum has been previously linked to punishment-based avoidance, with dorsal striatum damage resulting in suboptimal defensive choice.^47^ We situate our findings with consideration of this prior work and interpret Safety Meta-representation as necessary to generate choices about defensive action. The ventral striatum and hippocampus were both connected to the safety network during protection evaluation but not during threat evaluation. The vmPFC’s coupling with salience (insula) and reward regions (striatum) links self-relevant safety processing with affective and reward systems, suggesting its role in mediating the personal significance of stimuli and maintaining a sense of self across contexts.^48^ This is also consistent with our prior work demonstrating that safety conferred through protection is distinct from threat despite both occurring in aversive contexts^49^, likely due to its self-referential signaling.”***

***“Finally, our work is situated in a broader understanding that the vmPFC supports self-referential processing, extending this role to safety estimation. The brain is engaged in considerable focus on tracking associations related to the self.^31,32,35,46,48,69^ Our study demonstrates this does not just occur in conspecific social interactions or in identifying which objects belong to oneself, but also with respect to evolutionarily conserved threat appraisal. When the self is under threat, the vmPFC may help integrate threat-related stimuli into the broader narrative of self-representation, shaping responses based on past experiences and perceived safety. This raises a compelling case for considering how altered vmPFC activity in affective clinical disorders reflect disruptions in assessing and assigning personal significance to events, potentially tied to deficits in views of the self’s capacity to establish safety.”***

Minor

**R1.3.** It could be helpful for the figure legends to start with a more informative subtitle than 'neural activation' and 'behavioural results'. A brief summary of the main finding of each figure would be helpful for readers to grasp the main take home from the figure as an anchor for the longer legends. 

**A1.3. Thank you, we have expanded figure legend titles to start with a more informative heading to orient the reader to the more detailed legend information.**

***Figure 1. Safety Estimation Task and Safety Value Updating Task schematics, including safety probabilities, a conceptual model of safety estimation and integration, and MRI session procedure details.***

***Figure 2. Behavioral results showing subjects tracked safety probabilities but had biased predictions based on stimulus type and the order of stimulus presentation.***

***Figure 3. Neural response to safety increases during each task phase, highlighting the role of the ventromedial prefrontal cortex (vmPFC) in responding to safety increases and protection stimuli.***

***Figure 4. Multivariate functional network connectivity for trials of increased safety value versus those with increased danger value among a network of ten a priori selected regions of interest (ROIs) identified as relevant for threat/safety identification in the existing literature.***

***Fig S1. Neural response to danger increases during each task phase, highlighting regions of canonical defensive circuitry involved in threat detection such as the insula, thalamus, and PAG.***

***Fig S2. Results of stimuli development and selection of stimuli at the high and low ends of the safety estimation spectrum.***

**Reviewer #2:** Tashjian and colleagues submit a manuscript describing highly interesting and timely results form a study that combined a novel behavioral paradigm with fMRI. The study specifically examined whether the level of safety (e.g. having a weapon) influences the perception of a threat (e.g. dangerous animal) and further segregates the behavioral and neural subprocesses involved. The study is well designed, the manuscript is well organized, the results address an important gap in the literature and are cautiously interpreted. I have mainly minor points that may help to improve the manuscript further:

**We greatly appreciate the Reviewer’s support for this work and have addressed their comments below, which we believe have improved the manuscript.**

**R2.1.**     Authors discuss the background of the study and the results in the context of fear models. I wonder whether the design of the paradigm and the interpretation may also link with the Theory of Emotion from Lazarus, a cognitive emotion theory which underscorese the role of (re)appraisal (primary, secondary) in emotion processes. The level of evaluation that the authors operationalize in their paradigm is on a rather high cognitive evaluation level.

**A2.1. We agree that Lazarus’s Cognitive-Mediational Theory fits with our paradigm, suggesting that emotions arise from cognitive evaluations. However, it is difficult for us to empirically test this given our paradigm did not ask about emotions but rather only about cognitive appraisal (danger/safety), which is why we operationalize our paradigm in this cognitive evaluative level (in addition to evidence of vmPFC involvement in learning and cognitive appraisal under threat). We aren’t aware of any specific work linking Lazarus’s traditional theory of emotions to vmPFC activation, but it does align with the Somatic Marker Hypothesis (Damasio, 1994) that points to vmPFC involvement in integrating emotions and cognition. However, the specificity of SMH has been questioned so we have not interpreted our findings with respect to SMH specifically (see - Dunn, Dalgleish, Lawrence, 2006 Neuroscience & Biobehavioral Reviews). We now discuss the process of emotion/cognition interaction in the discussion and integrate it with work pointing to a role of the vmPFC in self-referential processing as raised by Reviewer 1:**

**“*Our findings converge with prior work indicating the vmPFC is involved in updating mental representations of threat and reward particularly in contexts like fear extinction.^17^ Given vmPFC involvement in the physiological regulation of autonomic processes like heart rate and skin conductance,^42,43^ it is likely that vmPFC engagement we saw during our task reflects a more complicated myriad of processes that we did not explicitly measure, including emotion regulation.^44,4545^ Additionally, our behavioral results show safety recognition varied as a function of stimulus type, and the vmPFC was preferentially activated to protection during the Safety Estimation Task. These findings are consistent with the vmPFC playing a role in self-relevant processes specifically rather than undifferentiated emotion regulation broadly. If the vmPFC were primarily tracking the latter, we would expect to see consistent activation regardless of stimulus type because both stimuli presentations occur during a need for regulatory engagement. Our findings fit with an interpretation of the vmPFC as providing a substrate for linking experiences, contexts, and events to the bio-regulatory or emotional state of the self.^32,46^”***

**R2.2.**       The vmPFC has been engaged in several functions related to fear and safety, but also associated phsyiological reactions such as regulation of cardiac reactivity etc. In their account the authors relate this region to meta-representation and updating. Authors may want to discuss or provide further analyses to differentiate the cognitive computations from the associated phsyiological reactivity or regulation.

**A2.2. We agree that the vmPFC has multiple functions, including those related to cognitive control (such as meta-representation and updating) and physiological regulation (such as cardiac reactivity) (see also the somatic-marker hypothesis). In our study, these functions can be dissociated to an extent based on the task demands - our task counterbalanced safety condition across animals and weapons and along the continuum with increasing safety value which should have equivalent contributions from vmPFC in terms of physiological response. We added additional text to the discussion to address the vmPFC role in physiological regulation during emotion regulation as well as threat-related cognition as noted above in A2.1.**

**R2.3.**       For the multivariate connectivity authors report in the text that results are presented on the level of "all connections p<0.05". Given the number of multivariate connections please clarify if multiple comparisons were applied.

**A2.3. Because IC analysis involves examining interactions between regions, the test looks at the structure of the whole network rather than isolated pairwise comparisons between individual regions. Network-based methods, such as betweenness centrality inherently aggregate across multiple connections, reducing the need for traditional correction for multiple comparisons and calling for permutation testing methods as laid out in the original methods paper (Coutanche & Thompson-Schil, 2013). As part of the IC toolbox used, we conducted permutation testing to determine the statistical significance of ROI-based IC analyses. We’ve now amended this to be more clear: “*Connections are reported at p<0.05, corrected based on within-subject permutation testing (10,000 iterations) as per the permute_ROI_IC() function within the IC Toolbox.^76^*”**

**R2.4.**       Authors report that (line 337) 'subjects fixated more on the initial safety value if protection stimuli were presented first…. ' however, it appears that the analyses in the behavioral and MRI sample were not consistent (p = .004, and p = .75). Please clarify and interpret the potential differences. 

**A2.4. Thank you for pointing this out – this was an error on our part in transposing results. At one point we fit computational models to address ‘stickiness’ in decisions (i.e., fixation to the initial safety value) with t-tests between model parameters for each sample. However, the models poorly converged and the sample size for the MRI sample was low, reducing reliability. We instead report results of the mixed effects logistic regression, which we’ve updated accordingly: “*B=.23, SE=.02, z=11.20, p<.001; MRI B=.15, SE=.03, z=4.04, p<.001), particularly when the protection information increased the likelihood of safety, (interaction between stimulus type and safety change: behavioral sample B=.04, SE=.006, z=6.84, p<.001; MRI sample B=.12, SE=.01, z=9.59, p<.001, Fig. 2c). Overall, subjects rated winning probabilities as higher if the second stimulus was a powerful weapon following a dangerous animal, compared to a weak weapon following a safe animal (75% versus 53%), despite equivalent experimentally established safety probabilities (Fig. 2d). However, subjects rated safety probabilities as equivalent when a dangerous animal followed a weak weapon or a safe animal followed a powerful weapon (63% for both) (Fig. 2e).”***

**We also clarified these results in the methods *“Behavioral Models. Psychometric curves were fit to examine safety prediction as a function of stimulus type (protection, threat) (Fig. 2b). α parameters represent the threshold level where safety estimation reached a 50% probability. General effect sizes are reported as 95% confidence intervals. Binary logistic mixed effects models were fit to examine whether (1) subjects tracked the experimentally established safety continuum with subjective estimations of winning and losing during safety prediction and safety meta-representation (Fig. 2a), (2) subjects modified safety estimation during safety meta-representation as a function of stimulus presentation order (protection first versus threat first) (not depicted graphically), and (3) safety change (increasing or decreasing from average) moderated the effect of stimulus presentation order on safety estimation modification (Model 2) (Fig. 2c). Mixed effects models were estimated using R (version 4.1.3) and the lme4 package (version 1.1.28).^72^ ^73^*”**

**We’ve confirmed all other behavioral results are correct.**

**We added additional plots to illustrate this effect to Figure 2c.**

**Fig. 2. (c)** Subjects were more likely to modify their safety estimations during *Safety Meta-representation* when threat stimuli were presented first followed by protective stimuli, especially when the safety value of the second stimulus changed to increase safety probability.

**R2.5.**       Authors observed that activation corresponding to decreases in safety was observed in the occipital pole and postcentral gyrus (in Fig. S1a-b). How could this be explained? Do these regions reflect preparatory visual and motor engagement to facilitate threat detection? 

**A2.5. We now discuss the interpretation of this finding in the discussion: “*As threat stimuli increased in danger during Safety Prediction, so did activation in the occipital pole and postcentral gyrus. We interpret this activation to reflect several processes related to visual, attention, and sensory processing when evaluating stimuli with heightened salience, like those representing a threat. The occipital pole is involved when visual processing demands are enhanced, as is the case when evaluating threatening stimuli that capture attention more effectively – as is shown in prior work suggesting threatening stimuli are prioritized in the visual system.^36,37^ The postcentral gyrus contains the primary somatosensory cortex and is involved in processing somatic sensory information, which may reflect an embodied response to perceived threat preparing for potential defensive action.^38^ Enhanced sensory processing in both visual and somatosensory areas may thus serve as an adaptive mechanism to improve threat detection and prepare for action.*”**

**We also added an interpretation of the findings with respect to activation for increasing threat during safety meta-representation to the discussion: “*As threat stimuli increased in danger during Safety Meta-representation, so did activation in defensive circuitry, including the insula, thalamus, ACC, and PAG. Parametric increases in activation of the thalamus, insula, pre-supplementary motor cortex, and medial occipital cortex suggest heightened sensory and motor preparation to manage escalating danger. This aligns with models of threat appraisal, where sensory processing regions and motor planning systems engage to anticipate defensive actions. These responses reflect a dynamic and context-sensitive process, consistent with the brain’s reliance on interconnected defensive circuits to adapt to changes in safety.”***

**Reviewer #3:** Tashjian and colleagues provide a novel approach to unpacking the neural dynamics of safety processes in the human vmPFC, combining behavioral and fMRI experiments to target understudied domains of safety encoding. Their findings appear to fit well with the known role of vmPFC in safety processing but also highlight more precise mechanistic contributions and differentiate discrete roles of vmPFC subregions (anterior vs posterior), and wider multivariate brain connectivity. I am impressed by the sophistication of the task - and the results are compelling. For these to be convincing, additional details on the study design, experiment methods, and analyses that should be provided. Conceptually, there ought to be clearer explanation of certain key concepts that are central to the work (e.g., meta-representation), which may not be familiar to other researchers who work on safety processing and the vmPFC and require greater contextualizing. I hope my comments below
are helpful in outlining these points.

**We appreciate the thoughtful suggestions from the Reviewer, which have been addressed below. The Reviewer’s comments were helpful in clarifying and contextualizing our study to make a broader and more accessible contribution. We also provide more details on design/methods/analyses.**

Major Comments:
**R3.1.**      The main strength of the work also presents some potential areas of concern.  Authors provide some novel and interesting angles to the safety processing literature, which I agree do well to complement existing models (e.g., avoidance/Pavlovian paradigms). However, the high novelty means that these concepts need to be well supported, defined, and justified. For example, phrases like 'meta-representation' may seem obscure to some. First, it is necessary (where applicable) to clarify the extent to which these kinds of constructs are entirely new - introduced here by the authors for the first time - or if they are using concepts that already exist in other research areas and leveraging these to gain a better model of safety-related neural processes.

**A3.1. We appreciate the Reviewer’s suggestion and have taken several steps to address this concern. We include a Key Concepts box in line with comment R3.12 below. We also now clarify in a few places related constructs of meta-representation such as replay/reactivation and discuss how our study differs:**

***“Key Concepts***

***Throughout we refer to several key concepts that are important to understand and interpret this work. The overarching investigation is to understand how humans estimate safety. Safety Estimation is the process by which humans evaluate the likelihood of safety (typically survival or absence of harm) in the presence of threats. Safety Estimation is central to understanding adaptive behaviors and defensive responses, and occurs through the integration of information about both external threat information and internal protective capacities. Safety Integration is the process of merging distinct pieces of information (e.g., sensory stimuli, prior knowledge, or contextual factors) to create a coherent and actionable representation. This work refers to the theory that safety is not solely determined by the sensory features of a threat but also by self-relevant factors. Meta-representation is a process that occurs during integration whereby the brain constructs an internal model of two (or more) pieces of information, updating the representation of one piece of information that is not immediately perceptible based on new information received. Meta-representation occurs during cognitive integration, where higher-order neural regions such as the prefrontal cortex and association areas combine sensory inputs with memories, predictions, and contextual cues. This concept aligns with several existing theories in cognitive neuroscience to explain how the brain achieves this complex task, including Bayesian Inference, Multisensory Integration, and Predictive Coding. Safety Recognition occurs when one can confirm safety where outcomes are known and Safety Value Updating occurs when one adjusts safety estimations based on new experiences or learning. Finally, we note that we refer to protective stimuli in this study as ‘self-relevant’. This characterization is in comparison to the threat information, which is fully external to the subject. We acknowledge that the vmPFC is implicated in a myriad of self-related processes (e.g., linking episodic memories to form coherent self-concepts, moral reasoning, and social comparisons). For the purposes of the present work, we consider stimuli to be ‘self-relevant’ when the value of a stimulus is reliant on how much a stimulus aids in fulfilling immediate, self-related goals, rather than on the stimulus itself.”***

**“*This process of reactivation/replay has been observed in aversive learning in humans.^34,39^ We observed patterns of activation at a whole-brain level, demonstrating striking similarities to the initial state encountered. We did not conceptualize meta-representation as replay in our study because our task does not afford the typical planning opportunity that is tested during traditional examinations of replay.*”**

**R3.2.**      Overall, the figures are useful, but the legends could be improved to clarify pertinent details. For example, Figure 1's legend should be able to explain each part of the figure - leaving no nomenclature unturned. Terms 'R1', R2, etc., are used, as is 'naïve', in parentheses, for certain areas of the figure. Please ensure there is no room for confusion, as understanding the experiment design in full helps understand the results. Most of the other figure legends have very comprehensive descriptions - which are appreciated.

**A3.2. We amended Figure 1 to remove nomenclature and present more fulsome details.**

**Figure 1. Safety Estimation Task and Safety Value Updating Task schematics, including safety probabilities, a conceptual model of safety estimation and integration, and MRI session procedure details. (a) Safety Estimation Task.** An example trial is presented. Subjects were told to imagine they were battling dangerous animals with powerful weapons. On each trial, subjects saw stimuli pairs comprised of a threat (animal) and protection (weapon) with presentation of threat/protection counterbalanced. First a weapon or animal was presented (*Safety Prediction*) and subjects made an initial estimation of whether they would win or lose the battle, responding with a button press. Then the paired stimulus was presented (*Safety Meta-representation*) and subjects made an updated judgment as to whether they would win or lose, responding with a button press. After both stimuli were presented, subjects saw the outcome of the battle depicted as either a shock (loss) or no shock (win) (*Safety Recognition*). If subjects lost and received the shock image, they had a 20% chance of receiving an electric shock to the wrist. **(b)** **Safety probabilities.** Threat and protection stimuli were each set on a four-point continuum with equivalent experimentally established safety probabilities. Italics depict the average shock value for each stimulus across all pairings. Paired probabilities are depicted in the heatmap. Probabilities were experimentally established prior to testing and were not made known to participants although the continua were easily identified based on prior knowledge and outcomes during the task. **(c)** **Model of safety estimation.** When only the first stimulus is known, *Prediction* (left) is biased and based only on the potency and safety value of the first stimulus presented. This partial information phase isolates variability in behavioral and neural response as a function of relevance (i.e., whether the first stimulus presented is an external threat or self-relevant protection). When the second stimulus is presented, subjects update safety estimates through *Meta-representations* (right) of the first stimulus (no longer perceptible) and new information about the second stimulus. This integration phase isolates variability in behavioral and neural response as a function of the order of presentation. Together, the Prediction and Meta-representation phases converge on a final estimate of safety based on the combined stimulus set. **(d)** **Safety Value Updating Task.** Blocks of stimuli were presented according to the safety value established for the Safety Estimation Task. Each stimulus was presented 5x in a block. Subjects were told to look at the stimuli carefully and that no judgments were to be made and no shocks would be delivered. **(e)** **MRI session procedure.** On the first day, subjects completed the naive version of the Safety Value Updating Task. At this point, subjects had not completed instructions for the Safety Estimation Task and thus had no knowledge of the task or stimuli relevance. Subjects then completed a structural scan, during which they learned about the Safety Estimation Task. After completing five practice trials, subjects completed Run 1 and Run 2 of the Safety Estimation Task. An average of 26 minutes elapsed from the start of Run 1 to the start of Run 2. On the second day, subjects completed Run 3 and Run 4 of the Safety Estimation Task, with an average of 25 minutes from the start of Run 3 to the start of Run 4. After Run 4 ended, subjects completed the knowledgeable version of the Safety Value Updating Task. Day 2 of testing took place on average 1 day and 7 hours after day 1.

**We have also amended the other figures to improve clarity and removed nomenclature from other figure legends (e.g., ‘BC’ -> ‘betweenness centrality’; ‘IC’ -> ‘Informational Connectivity).**

**In line with Reviewer 1’s comments we also added more detailed titles to the legends to orient the reader prior to the full legend details.**

***Figure 1. Safety Estimation Task and Safety Value Updating Task schematics, including safety probabilities, a conceptual model of safety estimation and integration, and MRI session procedure details.***

***Figure 2. Behavioral results showing subjects tracked safety probabilities but had biased predictions based on stimulus type and the order of stimulus presentation.***

***Figure 3. Neural response to safety increases during each task phase, highlighting the role of the ventromedial prefrontal cortex (vmPFC) in responding to safety increases and protection stimuli.***

***Figure 4. Multivariate functional network connectivity for trials of increased safety value versus those with increased danger value among a network of ten a priori selected regions of interest (ROIs) identified as relevant for threat/safety identification in the existing literature.***

***Fig S1. Neural response to danger increases during each task phase, highlighting regions of canonical defensive circuitry involved in threat detection such as the insula, thalamus, and PAG.***

***Fig S2. Results of stimuli development and selection of stimuli at the high and low ends of the safety estimation spectrum.***

**R3.3.**      More detailed reporting is required when outlining experiment task parameters, such as the length of the inter-trial/inter-stimulus intervals, and so forth, for all parts of the experiments. It should not be too onerous to add seconds/milliseconds in parentheses wherever applicable in-text for the methods sections. I hasten to note that the authors ought not to place too much reliance in figures or in-text descriptions exclusively, the methods should provide all relevant details, and these can be illustrated in figures as a useful adjunct, but ultimately, critical parameters ought not be missing in-text. Other examples of critical information include stimulus durations, how these varied in sync with behavioral responses (if they did), and the time gaps/breaks between task phases (even if approximate). It is a very novel and idiosyncratic set of tasks, and if one sought to replicate/translate the tasks, these details are critical.

**A3.3. We agree that given these are novel tasks more detail is needed and have added parameters for the tasks in the Methods while also retaining details in the figures.**

**“*For each trial, subjects saw 2 images presented for up to 6 seconds maximum. Image presentation offset with subject response. Each image was separated by an interstimulus interval (ISI) presented for 2-5seconds with duration jittered. Each pair of images contained one weapon and one animal, with the presentation order counterbalanced. Subjects were told to indicate with a button press while the stimulus was on screen whether they thought they would win or lose the battle against the animal with the weapon provided. Subjects used their right index finger to indicate a win prediction and their right middle finger to indicate a loss prediction. As soon as predictions were made the stimulus offset and the ISI was presented. Subjects were told “winning does not necessarily mean killing the other animal. You can interpret winning as defeating the other animal either because it retreats or because it is physically defeated”. Response to the first image presentation was based on partial information whereas response to the second image presentation was based on full information of the animal/weapon pair. Animals and weapons ranged in safety value on a 4-point continuum with matched contingencies (Fig. 1b). Likelihood of win/loss depended on the combined probability of the animal/weapon. For example, if subjects encountered a lion, they had an average 64.29% likelihood of losing the battle regardless of the weapon. That likelihood increased to 78.57% if subjects were equipped with a stick and reduced to 42.86% if subjects were equipped with a grenade. After both images were presented, subjects saw the outcome of the battle for 2 seconds. For the MRI sample, subjects had a 20% chance of receiving an electric shock to the wrist for every lost battle. Subjects were 100% safe from electric shock if they won the battle. For the behavioral sample, points were lost and gained depending on battle outcomes. Trials were separated by an inter-trial interval (ITI), presented for 1-6 seconds with duration jittered. All subjects completed 448 trials. The behavioral sample completed trials in a single session whereas the MRI sample completed 4 runs of 112 trials each run each over 2 days. The first two runs were presented on day 1, with an average of 26 minutes from the start of Run 1 to the start of Run 2. The second two runs were presented on day 2, which occurred on average 1 day and 7 hours after day 1, and the time between the start of Run 3 and the start of Run 4 was 25 minutes on average. The Safety Estimation Task was programmed using PsychoPy v2021.2.3.”***

**R3.4.**      As per above, please reconcile/explain using terms ITI and ISI (intertrial and interstimulus intervals) to describe a similar thing in the same paragraph (page 25).

**A3.4. While both ITI and ISI describe time gaps in the task paradigm, the key difference is that ITI deals with the time between entire trials, whereas ISI is concerned with the timing between individual stimuli within a trial. Both intervals are important for minimizing confounds and allowing for accurate measurement of neural responses. We now clarify that “*Images were presented for .5 seconds per image. Images were presented 5x in each block. Six blocks were repeated 5x each. Each stimulus image was followed by a 3 second ISI (interstimulus interval breaking up the presentation of individual stimuli) and each 20-trial set within a block was followed by a 12-second ITI (intertrial interval breaking up entire blocks). This approach was selected as it has been shown to be a validated approach for the localization of neural regions specifically responsive to stimuli recognition.^71^ Blocks were comprised of high danger stimuli with high shock probability during the Safety Estimation Task, high safety stimuli with low shock probability during the Safety Estimation Task, high threat stimuli with high external threat value outside of the experimental environment, low threat stimuli that have low external threat value outside of the environmental experiment, low threat stimuli with low external threat value, weapons, and animals.” (full cite: Fox, C. J., Iaria, G. & Barton, J. J. Defining the face processing network: optimization of the functional localizer in fMRI. Human brain mapping 30, 1637-1651 (2009))***

**R3.5.**      The methods for designing stimuli for proper validity appear highly commendable and the detail in reporting is appreciated. Firstly, if there is any available data as to the choice of stimulus design, this could be helpful in supplementary material. Secondly, concerning post-task checks and balances, I would be interested to know if authors assured construct validity of the tasks, in terms of participants having the intended experiences. For instance, on what basis do authors feel confident that they successfully evoked and manipulated thoughts/feelings/behaviors that were selective to the construct of 'safety' versus other affective states (or merely nonaffective processing)? At a basic level, can we be confident in assuming that this study effectively evoked active safety-specific processes, rather than non-emotional/natural stimulus processing/estimations? Finally, the animals and defensive items are likely to have varied in terms of inherent pleasant/unpleasant/anxiety-arousing connotations (e.g., domestic cats compared to charismatic megafauna, or sticks compared to guns/knives). To be clear, I am strongly convinced by behavioral data (Fig2) that the stimuli and task worked well for computing the probabilities of safety/threat. Though not directly central, I wonder if the affective value (prior to any learning) may be an interesting component to prediction/estimation/meta-representation.

**A3.5. We agree affective dynamics are interesting. We note that we did not set out to measure ‘fear’ as an emotional response, which is why we keep the language in the manuscript tied to safety and threat as more cognitive constructs that evoke a defensive response (see LeDoux, 2014 *PNAS*).**

**We have several reasons to believe the construct validity in terms of safety and threat cognition was met. The inclusion of analyses with respect to the outcome phase of the trial provides a robust check for evoking safety response. During the outcome subjects are certain as to whether they won or lost the battle, with losing battles running the risk of shock. As shown in Figure 3h, ‘win’ trials resulted in activation of the striatum and hippocampus – commonly associated with reward/safety – whereas in Figure S1g, ‘lose’ trials resulted in activation of the insula and PAG – commonly associated with danger/threat. We agree the stimuli were not controlled on all dimensions (unpleasantness, anxiety arousal) but as the Reviewer noted our behavioral findings and stimuli development responses indicate they did sufficiently on the dynamic of interest (safety/danger - ratings during stimuli development, which are provided in Supporting Information Figure S2). The safety localizer task was intended to test neural response to the affective value of the stimuli prior to learning about their specific value to the subjects. As shown, we see changes in vmPFC activation from post to pre-task.**

**In line with suggestions by the Reviewer, which we agree are interesting, we discuss in the limitations that further inquiries into the subjective experience of the task and the stimuli presented would strengthen conclusions and be useful for future work: “*Third, how do affect dynamics influence safety estimation? We did not collect participant report on their affective response to stimuli during the task. As a result, we cannot be certain whether our findings reflect an affective or non-emotional component of safety processing. Other work indicates a role of the vmPFC in the extinction of emotional arousal,^56^ and future work should consider how that role generalizes to safety estimation.*”**

**We also include more information about the stimuli design as requested in supplemental materials as part of Figure S2. “*Fig S2. Results of stimuli development and selection of stimuli at the high and low ends of the safety estimation spectrum. Two questions were asked related to level of danger (animals) and power (weapons) of 20 potential stimuli images. Items were also paired in head-to-head battles with all other stimuli of the same type. Lion and grizzly were rated as the most dangerous stimuli and cat and goose were rated as the second and third least dangerous stimuli (rat was selected as the least dangerous but ultimately excluded from the set to avoid conflating threat with disgust). The same rankings were reported for the head-to-head battles across all animals. The grenade and gun were rated as the most powerful weapons and as most likely to win head-to-head. Fist and stick were rated in the bottom 30% of power ratings and bottom 20% of head-to-heads. Other weapons rated as less powerful were excluded due to concerns of unwieldy usage (i.e., rope). Simulus design was inspired by 2021 YouGov survey of 1224 adults showing that that 6% of Americans believe they could beat a grizzly bear in a fight without weapons (retrieved from: https://today.yougov.com/society/articles/35852-lions-and-tigers-and-bears-what-animal-would-win-f). Animals were selected as pilot stimuli based on those survey results and a selection of weapons across the range of potential danger was also tested. Animal images were selected to be ‘attacking’ to mitigate any issues of liking the animal (cuddly housecat versus angry housecat) to ensure stimuli were immediately recognizable as a threat. Weapon images were selected to be without any other stimuli in the picture and on white backgrounds. All images were kept in black and white and sepia tone ranges.*”**

**R3.6.**      Related to the above comment, do authors confidently assert that there was/wasn't some element of associative learning occurring/interacting with their intended effects? It is hard to avoid the possibility that partial aversive reinforcement (20% shocked 'lost battle' trials) may have induced learning effects. Since the task seems to emphasize inherent properties of stimuli (e.g., assumed knowledge about the efficacy of handguns vs sticks, or danger of house cats vs lions), is there a potential influence of shock-induced associative fear/safety learning? If this is a large factor, then how much can it truly be said the task differs from 'external' threat/safety tasks (e.g., fear conditioning/extinction).

**A3.6. We do anticipate that participants learned to associate stimuli with a probability of shock to a certain extent. However, rather than associating a particular stimulus with a shock, the shocks occurred on a random basis (20% of trials where subjects lost the battle) to provide an association between lost battles and shocks. Further, a crucial purpose of this task design was to use inherently dangerous stimuli and stimuli with a clear distinction between high and low danger, such as cats versus lions or handguns versus sticks. Stimuli with well-established associations with danger, such as handguns and lions, should activate neural circuits related to threat detection as a function of their inherent properties (See Dunsmoor et al., 2011). This reduces the likelihood that shock-induced fear conditioning would significantly alter how these stimuli are perceived. Because the danger of the stimulus is already “coded” as part of its inherent properties, new associations are attenuated and the brain response is relatively predictable (in line with predictive coding theories; Friston, 2005).**

**We note that associative learning forms the foundation of the understanding that certain stimuli are dangerous and that the vmPFC is likely representing that associative value at the time of choice. However, we don’t conceptualize this task as an associative learning task for the reasons laid out above.**

**We now include this rationale in the discussion “*Battle outcomes were tested as Safety Recognition and served as the purest test of safety neural circuitry. Neural activation in response to safety certainty, when subjects won the battle and were 100% safe from shock compared to when they lost battles, increased in the vmPFC, striatum, and hippocampus. In response to lost battles when there was risk of electric shock, compared with won battles, subjects demonstrated increased engagement of canonical defensive circuitry in the PAG and bilateral insula. Activation of the striatum and hippocampus indicates subjects learned during outcomes but did not reengage these circuits during prediction. The vmPFC, however, was engaged in response to both Safety Prediction and Recognition. We interpret the difference in vmPFC engagement compared with striatum and hippocampus engagement to indicate a more general role of the vmPFC in recognizing and predicting safety, rather than tracking outcomes to reinforce learning. This interpretation is further supported by using stimuli that had known and biologically relevant danger values (predators and weapons), which likely attenuates online associative learning during the task because the danger of the stimulus is already coded as part of its inherent properties.^52^ The brain should treat these stimuli as known^53^ and, therefore, vmPFC activation is unlikely a result of generating new learning effects in response to incidental shock pairings.*”**

**R3.7.**      The 'objective' underlying probabilities of threat, concerning the different combinations of animals + defense strategies (e.g., Fig.1C) is an appealing means for ecological validity in the task (combining a bear and gun = higher % of safety versus combination of bear and stick). Since these are 'objective' and represented in discrete numerical values (64.29%, 78.57%, as examples), can authors please clarify their methods for deriving said percentages? This was a little unclear to me on first reading. The term 'objective' may also raise eyebrows. How do we deliberate on the issue of whether a bear + gun situation is objectively more/less safe than a bear + hand grenade scenario? Is the room for variability here a feature or a bug?

**A3.7. We agree and although we like the language ‘objective’ for ease of interpretation have changed this to ‘experimentally established’ throughout the manuscript. We also further discuss this decision in the Methods of the task: “*We experimentally established safety probabilities to control comparisons between types of stimuli (threat versus protection) along the safety continuum, but we acknowledge that these probabilities are not definitive or universally applicable outside of the experimental setting.*”**

**R3.8.**      Details are provide concerning what was performed for each group, but I would appreciate clarification on why. For the task features present/absent in behavioral/fMRI groups, please explain reasons for there being a difference, the purpose of said differences.

**A3.8. The behavioral and MRI subjects performed the same Safety Task, with the only difference being that the fMRI group performed the task in the scanner on two different days. We now clarify in the methods:**

**“*One hundred thirteen human subjects completed the Safety Estimation Task online, which was identical to the task performed in the MRI scanner except that it was performed in a single continuous session for the Behavioral subjects. The Behavioral subjects did not complete the Safety Value Updating Task because there were no decision components for this task, so no meaningful data could be collected.”***

***“ Thirty-one human subjects completed the same Safety Estimation Task as the Behavioral sample. The only difference between the Behavioral and MRI samples is that the MRI sample completed the Safety Estimation Task in four blocks over two days while undergoing functional MRI. Separating the MRI sessions into two days was designed to alleviate motion problems from subjects spending too long in the scanner without a break. MRI subjects also completed the Safety Value Updating Task described below.*”**

**R3.9.**      Briefly, given the high spatial and temporal resolution of the fMRI acquisition (for a 3Tesla scanner, settings seem to have a combination of relatively small voxels + fast TR), please explain confidence in the quality of imaging data. What head coil (e.g., number of channels) was used? Did fmriprep provide useful quality checks that validated basic levels of data quality? Additionally, do the authors consider accelerated resolution of this kind to be a generally applicable approach or are these settings tuned to be sensitive to their specific study needs?

**A3.9. Our TR = 1.12 s is within the typical range for fMRI acquisitions as standard TR values typically range from 1.0 to 2.5 seconds depending on resolution and scan time requirements. The choice of a 1.12 s TR allows for higher temporal resolution and is appropriate for capturing faster hemodynamic responses, especially in tasks involving rapid events.**

**Our TE = 30 ms is also typical for fMRI with a T2* weighted sequence. Standard TEs for fMRI typically range from 25 ms to 40 ms, depending on the trade-off between spatial resolution and sensitivity to BOLD contrast. Shorter TEs (closer to 30 ms) are preferred to minimize signal loss from the dephasing of spins, especially in regions with inhomogeneous magnetic fields such as the vmPFC with air-tissue boundaries (sinuses).**

**Our voxel size of 2 x 2 x 2 mm is considered high resolution for functional imaging. Typical voxel sizes for fMRI are generally in the range of 2 x 2 x 2 mm to 4 x 4 x 4 mm. Higher resolutions, like 2 mm isotropic, provide more detailed anatomical localization of brain activity but can reduce the signal-to-noise ratio (SNR) because of the smaller volume of tissue per voxel. Our voxel size is consistent with high-quality fMRI datasets, especially when combined with the multiband acceleration factor.**

**Our multiband factor of 4 is a form of parallel imaging that speeds up data acquisition by simultaneously collecting data from multiple slices. This is quite common in modern fMRI to reduce the temporal lag between slices and allow for faster TRs without sacrificing spatial resolution. A factor of 4 is a moderate acceleration that typically provides a good balance of speed and image quality according to Setsompop, K., et al. (2012). Multiband MRI imaging techniques. *NeuroImage*, 62(2), 612-623, which states ‘We demonstrate the effectiveness of multiband acceleration factors of 2–8, with factors of 4 being commonly used to balance spatial and temporal resolution while maintaining acceptable signal-to-noise ratios (SNR).’**

**We used a 32-channel head coil which is now referenced in the ‘MRI Data Acquisition’ section.**

**fMRIprep provided checks for motion, SNR, and global signal, all of which confirm the acquisition of high-quality data.**

**As for the general applicability of the resolution settings, the specific acquisition parameters are standard parameters used at our scan center and while we believe they are suitable for similar tasks to balance spatial and temporal resolution, we do not consider them to be universally optimized for all fMRI applications.**

**R3.10.**     I became a little lost looking between the results, methods, and figures concerning how many ROIs were included and which analyses these were each applied to. Based on its current form, the in-text methods suggest 1 vmPFC ROI, but it also there are at least 2 as per the multivariate connectivity results (Fig.4, for example). I believe the use of multivariate searchlight analysis may obscure things. For readers grounded in more conventional fMRI approaches, clarification is needed on which results were whole-brain, ROI, or where regions were defined based on searchlight search within an ROI (or indeed across whole brain if this was done). To be specific, there needs to be clarity on how authors identified one area of voxels as being region X, and another as region Y. There are phrases in some parts of the paper that suggest whole brain searchlight identified the vmPFC, others that indicate that searchlight was more targeted. Wording must remain consistent - as to whether activations in certain areas were discovered incidentally or sought out explicitly.

**A3.10. ROIs were only applied to the informational connectivity analyses. A small volume correction in the vmPFC (ROI defined by Neurovault) was conducted for multivariate searchlight to constrain search space. All other analyses were whole brain. We clarify this in the methods section ‘Regions of Interest (ROIs)’: “*ROIs were used in two analyses, multivariate searchlight, and multivariate informational connectivity. All other analyses were conducted using the whole brain. ROIs were independently defined based on analytic purposes. First, a large vmPFC mask was independently defined using Neurovault and applied as a small-volume correction for the Safety Value Updating searchlight analysis. The vmPFC mask (***[***https://identifiers.org/neurovault.image:132836***](https://identifiers.org/neurovault.image:132836)***) consisted of 152 subjects and 4233 voxels. The mask was transformed to standard space (MNI152NLin2009cAsym) using ‘flirt’ [FSL 6.0.5.1:57b01774, @flirt]. A large vmPFC mask was used to assess whether different subregions of the vmPFC were implicated in Safety Value Updating rather than constraining search to a specific subregion. Second, ten separate ROIs were defined for Multivariate Informational Connectivity analyses. Regions were selected based on threat acquisition and extinction circuitry in prior literature and defined using the Harvard-Oxford cortical and subcortical structural atlases, except for the PAG which was defined using Neurosynth meta-analysis (https://neurosynth.org/; “periaqueductal” with an association test). Instead of using the larger homogenous vmPFC structure as constrained during searchlight, we divided the vmPFC into anterior and posterior subparts based on the Harvard-Oxford atlas to examine distinct connectivity within these regions. The vmPFC ROIs used in Informational Connectivity analyses were contained within the vmPFC mask used in the searchlight. The ten ROIs selected were the anterior vmPFC (‘frontal medial cortex’), posterior vmPFC (‘subcallosal cortex’), thalamus, ACC, bilateral insula, dorsal striatum, ventral striatum, PAG, bilateral amygdala, bilateral hippocampus.”***

**We also clarify that we conducted whole-brain analyses in the methods section ‘Univariate Analysis’: “*All univariate group-level fMRI analyses were conducted at the whole-brain level and corrected for multiple comparisons using FSL Randomise with 5000 permutations.*”**

**In line with Reviewer 1’s comment, we now include additional information mapping our results to specific anatomical boundaries. “*Safety activation in the vmPFC is primarily identified in area 14m with safety updating and recognition (conditions during which safety was guaranteed) extending to area 10 (frontal pole).^34^* “*The vmPFC coded safety during all task states from Safety Prediction to Meta-representation to Safety Recognition and Value Updating. Activation to these states was primarily identified in area 14m, with Safety Recognition and Value Updating extending to area 10. We identified what appeared to be a gradient from the posterior to the anterior part of vmPFC area 14m, with activation extending more anterior as safety increased in certainty. This apparent gradient supports the possibility of subparcellation of area 14m into smaller areas with posterior and anterior distinction. During detailed mapping of vmPFC architecture, Mackey & Petrides (2014) note that “the posterior part of area 14m is less well developed and is less granular than the anterior part”.^34^ This aligns with our recent proposition^2^ that the posterior vmPFC may encode simpler representations of threat whereas the anterior vmPFC may encode more complex safety associations. This apparent gradient aligns with the broader organizational gradient of the prefrontal cortex, where anterior regions are generally more granular and complex, reflecting their role in higher-order cognitive and integrative functions. Our findings support assertions that the anterior vmPFC plays a role in integrating the value of self-relevant stimuli to influence the higher-order construction of affective processes, including safety.^2,61^”***

**R3.11.**     Some areas use mathematical notation or symbols in ways that needlessly complicate simple things. Specifically, conjunction analyses. Figure 3's legend includes "Conjunction analyses for the safety prediction increasing safety > increasing danger ∩ safety meta-representation increasing safety > increasing danger for all stimuli, with overlapping activation in the vmPFC; Z=2.3, p<.05." which may be missing something. It would doubtless be easier to say there was a conjunction in activation/a shared activation in vmPFC between the two contrasts?

**A3.11. We changed this legend as follows: “*(g) Conjunction of Shared Activation between Safety Prediction and Safety Meta-representation. Conjunction analyses for the safety prediction phase of increasing safety versus increasing danger and shared activation with the safety meta-representation phase of increasing safety versus increasing danger. All stimuli represented. Results indicate overlapping activation in the vmPFC; Z=2.3, p<0.05.”***

**We also removed mathematical notation from other legends: *“(i) Safety Value Updating. Multivariate searchlight revealed neural activation change in the vmPFC when subjects engaged in Safety Value Updating for the high safety block (see Fig. 1d). Analyses examined the contrast of the Knowledgeable version (post-task viewing) versus the Naive version (pre-task viewing) (Fig. 1e). Searchlight was a priori restricted to the vmPFC using an ROI defined via Neurovault.*”**

**R3.12.**     In Discussion section, the following sentence presents some obstacles to easy interpretation: "This study identifies neural systems involved in safety coding, provides evidence that Safety Prediction evokes dissociable circuits depending on whether the stimulus has self-relevance, and supports the hypothesis that the brain integrates threat and protective information to Meta-represent safety.". As with some other parts of the paper, there is perhaps too much assumed knowledge on the part of the reader as to knowing (for example) what it means 'to meta-represent' something, or what the authors are referring to by 'self-relevance' in the context of their current task. The latter is particularly important since the vmPFC is so closely linked with self-related processes - which are often very different from the authors' task.  This comment is not specific to this sentence however, it may only require one area of the manuscript where such concepts or framings are precisely defined.

**R3.12.** **We have now added a ‘Key Concepts’** **section to the introduction. We believe this reference for readers will make the manuscript more accessible.**

*“****Key Concepts***

***Throughout we refer to several key concepts that are important to understand and interpret this work. The overarching investigation is to understand how humans estimate safety. Safety Estimation is the process by which humans evaluate the likelihood of safety (typically survival or absence of harm) in the presence of threats. Safety Estimation is central to understanding adaptive behaviors and defensive responses, and occurs through the integration of information about both external threat information and internal protective capacities. Safety Integration is the process of merging distinct pieces of information (e.g., sensory stimuli, prior knowledge, or contextual factors) to create a coherent and actionable representation. This work refers to the theory that safety is not solely determined by the sensory features of a threat but also by self-relevant factors. Meta-representation is a process that occurs during integration whereby the brain constructs an internal model of two (or more) pieces of information, updating the representation of one piece of information that is not immediately perceptible based on new information received. Meta-representation occurs during cognitive integration, where higher-order neural regions such as the prefrontal cortex and association areas combine sensory inputs with memories, predictions, and contextual cues. This concept aligns with several existing theories in cognitive neuroscience to explain how the brain achieves this complex task, including Bayesian Inference, Multisensory Integration, and Predictive Coding. Safety Recognition occurs when one can confirm safety where outcomes are known and Safety Value Updating occurs when one adjusts safety estimations based on new experiences or learning. Finally, we note that we refer to protective stimuli in this study as ‘self-relevant’. This characterization is in comparison to the threat information, which is fully external to the subject. We acknowledge that the vmPFC is implicated in a myriad of self-related processes (e.g., linking episodic memories to form coherent self-concepts, moral reasoning, and social comparisons). For the purposes of the present work, we consider stimuli to be ‘self-relevant’ when the value of a stimulus is reliant on how much a stimulus aids in fulfilling immediate, self-related goals, rather than on the stimulus itself.****”*

Minor Comments:
R3.13.     Page 3, line 57 "reducing stress, and initiating in other survival" - typo - the 'in' is probably not meant to be there

**A3.13. We removed ‘in’.**

**R3.14.**     Page 3 line 67: "…relevant information changes so can the safety estimate even if the…" - a comma needs to separate 'estimate' and 'even' I think.

**A3.14. We added a comma between ‘estimate’ and ‘even’.**

**R3.15.**     In some figures the brain activation images that are surrounded by small grey borders that appear to be unintended, perhaps an issue with converting file types. It should be fixed in next submission. Likewise, Figure 3 panel J seems to be showing some errors, half the axial slice is cut off (assuming this is not intentional).

**A3.15. We also see the grey border artifact in the preprint, which must have been due to file conversion. We have now amended the figure files which should resolve the issue. The axial slice cut off in Figure 3 panel J was intentionally to highlight the vmPFC with a larger resolution, but we’ve now replaced Figure 3 panel J with a full slice and have circled the vmPFC for emphasis.**
